# Supplementary material for: Revealing Pan-Histology Immunomodulatory Targets in Pediatric Central Nervous System Tumors
Source: Cancers (Basel). 2023 Nov 17;15(22):5455. doi: 10.3390/cancers15225455 (PMC10670190; doi:10.3390/cancers15225455)

| | Low-grade C3, PFS | | | | Low-grade C4, PFS | | | | High-grade C4, OS | | | |
|---|---|---|---|---|---|---|---|---|---|---|---|---|
| **Characteristic** | **N** | **HR**[1] | **95% CI**[1] | **p-value** | **N** | **HR**[1] | **95% CI**[1] | **p-value** | **N** | **HR**[1] | **95% CI**[1] | **p-value** |
| t cells | 80 | 0.60 | 0.38, 0.95 | **0.030** | 104 | 1.35 | 1.00, 1.83 | **0.047** | 239 | 1.27 | 1.05, 1.52 | **0.013** |
| macrophages | 80 | 1.01 | 0.98, 1.05 | 0.4 | 104 | 1.00 | 0.97, 1.02 | 0.9 | 239 | 0.98 | 0.97, 0.99 | **0.003** |
| neutrophils | 80 | 1.01 | 0.99, 1.02 | 0.5 | 104 | 0.99 | 0.95, 1.02 | 0.4 | 239 | 0.99 | 0.96, 1.02 | 0.5 |
| nK cells | 80 | 0.59 | 0.23, 1.47 | 0.3 | 104 | 1.19 | 1.03, 1.37 | **0.020** | 239 | 0.97 | 0.92, 1.03 | 0.3 |
| b cells | 80 | 2.48 | 0.89, 6.94 | 0.083 | 104 | 0.50 | 0.17, 1.43 | 0.2 | 239 | 0.83 | 0.54, 1.27 | 0.4 |
| proliferation | 80 | 1.14 | 0.81, 1.59 | 0.4 | 104 | 1.40 | 1.09, 1.81 | **0.008** | 239 | 1.02 | 0.99, 1.04 | 0.15 |
| Resection | 80 | | | | 104 | | | | 239 | | | |
| Biopsy | | — | — | | | — | — | | | — | — | |
| GTR | | 0.21 | 0.02, 2.21 | 0.2 | | 0.05 | 0.01, 0.37 | **0.003** | | 0.40 | 0.20, 0.78 | **0.007** |
| Partial | | 0.45 | 0.04, 4.82 | 0.5 | | 0.42 | 0.07, 2.67 | 0.4 | | 0.64 | 0.37, 1.11 | 0.11 |
| Diagnosis | | | | | | | | | 239 | | | |
| ATRT | | | | | | | | | | — | — | |
| CPC | | | | | | | | | | 0.56 | 0.12, 2.65 | 0.5 |
| DMG | | | | | | | | | | 2.92 | 1.28, 6.66 | **0.011** |
| Embryonal | | | | | | | | | | 0.24 | 0.07, 0.83 | **0.024** |
| Ependymoma | | | | | | | | | | 0.20 | 0.08, 0.52 | **<0.001** |

| | Low-grade C3, PFS | | | | Low-grade C4, PFS | | | | High-grade C4, OS | | | |
|---|---|---|---|---|---|---|---|---|---|---|---|---|
| **Characteristic** | **N** | **HR[1]** | **95% CI[1]** | **p-value** | **N** | **HR[1]** | **95% CI[1]** | **p-value** | **N** | **HR[1]** | **95% CI[1]** | **p-value** |
| HGG | | | | | | | | | | 0.83 | 0.39, 1.73 | 0.6 |
| Medulloblastoma | | | | | | | | | | 0.26 | 0.13, 0.53 | **<0.001** |
| Sarcoma | | | | | | | | | | 0.42 | 0.12, 1.44 | 0.2 |

[1]HR = Hazard Ratio, CI = Confidence Interval

**Supplementary Table S1**: Figure 2A full model results demonstrating the HR of the $\log_2$ increase of *Imsig* signature among primary pCNS neoplasms. Results from three separate cox regression models are displayed. Low-grade C3 included N = 80 samples (18 observations deleted due to missingness) with 18 events. Low-grade C4 included samples N = 104 (41 observations deleted due to missingness) with 30 events. High-grade C4 included N = 239 samples (47 observations deleted due to missingness) with 127 events. Low-grade models examined impact of *Imsig* signatures on progression free survival, adjusted for extent of surgical resection. Addition of diagnosis code to the models resulted in failed convergence due to sample size constraints. High-grade C4 model is adjusted for extent of surgical resection and diagnosis code.

# Western blots

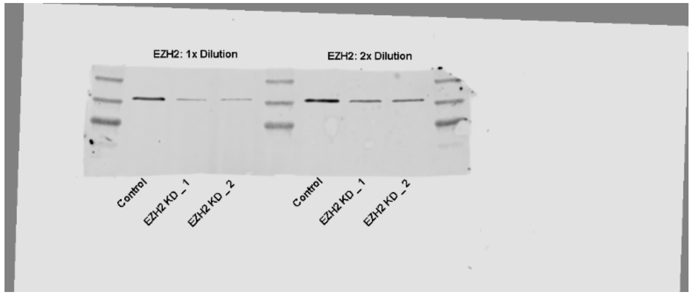


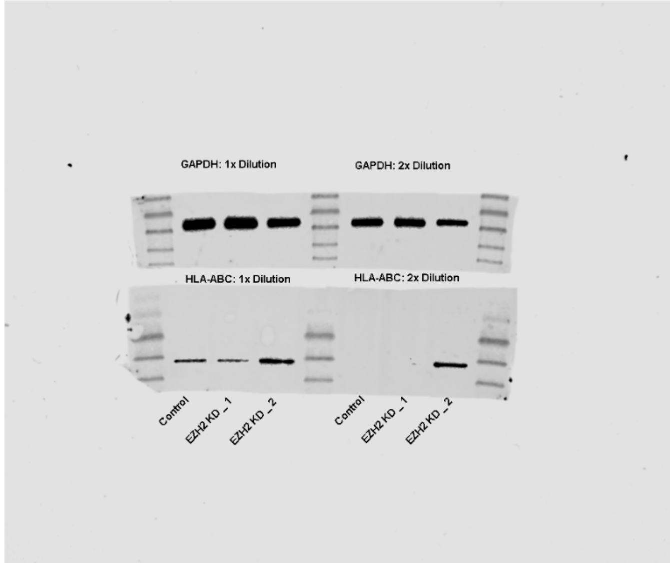

Supplement: Supplementary file 1 [file cancers-15-05455-s001.zip › PBTAManuscript_SupplementaryTable1.pdf]
